# A microarray-based system for the simultaneous analysis of single nucleotide polymorphisms in human genes involved in the metabolism of anti-malarial drugs

**DOI:** 10.1186/1475-2875-8-285

**Published:** 2009-12-09

**Authors:** Eva Maria Hodel, Serej D Ley, Weihong Qi, Frédéric Ariey, Blaise Genton, Hans-Peter Beck

**Affiliations:** 1 Swiss Tropical Institute, Socinstrasse 57, PO Box, 4002 Basel, Switzerland; 2 Institut Pasteur in Cambodia, Phnom Penh, Cambodia; 3 Department of Ambulatory Care and Community Medicine, University of Lausanne, Lausanne, Switzerland; 4 Papua New Guinea Institute of Medical Research, Goroka, Papua New Guinea; 5 Functional Genomics Center Zurich, Zurich, Switzerland

## Abstract

**Background:**

In order to provide a cost-effective tool to analyse pharmacogenetic markers in malaria treatment, DNA microarray technology was compared with sequencing of polymerase chain reaction (PCR) fragments to detect single nucleotide polymorphisms (SNPs) in a larger number of samples.

**Methods:**

The microarray was developed to affordably generate SNP data of genes encoding the human cytochrome P450 enzyme family (*CYP*) and *N*-acetyltransferase-2 (*NAT2*) involved in anti-malarial drug metabolisms and with known polymorphisms, i.e. *CYP2A6, CYP2B6*, *CYP2C8*, *CYP2C9*, *CYP2C19*, *CYP2D6*, *CYP3A4*, *CYP3A5*, and *NAT2*.

**Results:**

For some SNPs, i.e. CYP2A6*2, CYP2B6*5, CYP2C8*3, CYP2C9*3/*5, CYP2C19*3, CYP2D6*4 and NAT2*6/*7/*14, agreement between both techniques ranged from substantial to almost perfect (kappa index between 0.61 and 1.00), whilst for other SNPs a large variability from slight to substantial agreement (kappa index between 0.39 and 1.00) was found, e.g. CYP2D6*17 (2850C>T), CYP3A4*1B and CYP3A5*3.

**Conclusion:**

The major limit of the microarray technology for this purpose was lack of robustness and with a large number of missing data or with incorrect specificity.

## Background

Drug action depends on how drugs are metabolized and differences in activity of metabolizing enzymes can significantly contribute to the efficacy of drugs [1,2]. This might also be true for drugs given to treat malaria. The objective was to analyse single nucleotide polymorphisms (SNPs) in genes encoding enzymes implicated in metabolizing anti-malarial drugs in order to determine the contribution of these enzymes to the pharmacokinetics of the specific drugs. There are many methods available for the detection of SNPs (for review see [3,4]). These methods are either based on allele-specific hybridization or on primer extension reaction. Many of these techniques are time consuming, expensive and/or not suitable for use in resource-poor countries. Previously, a cost-effective DNA microarray-based [5] technique to detect SNPs associated with drug resistance in malaria parasites in a larger sample size has been developed and successfully used. To test whether this system also could be used for SNP determination in metabolizing enzyme genes, microarray determined SNPs were compared with sequencing. For this, a microarray was developed to affordably generate SNP data on genes encoding the human cytochrome P450 enzyme family (*CYP*) and *N*-acetyltransferase-2 (*NAT2*) involved in anti-malarial drug metabolisms. The performance of this microarray had to be determined subsequently. The microarray was designed to analyse enzyme genes with known polymorphisms such as *CYP2A6 *and *CYP2B6 *for the artemisinins, *CYP2C8 *for amodiaquine, chloroquine and dapsone, *CYP2C9 *and *NAT2 *for dapsone and sulphamethoxazole, *CYP2C19 *for dapsone and proguanil, *CYP2D6 *for chloroquine and halofantrine, *CYP3A4 *for the artemisinins, chloroquine, dapsone, halofantrine, lumefantrine, mefloquine, primaquine and quinine, and *CYP3A5 *for artemether, *β*-arteether, chloroquine, mefloquine, quinine and sulphadoxine [6-29]. For certain anti-malarial drugs (piperaquine, pyrimethamine and pyronaridine) the metabolic pathway is yet not well known, while others (atovaquone and doxycycline) are barely metabolized at all [30-35].

## Methods

### Study population

During an *in vivo *drug efficacy study in patients with uncomplicated malaria of all age, venous blood samples (anticoagulated using EDTA) were obtained after informed consent from 125 patients in Northern and Western Cambodia (64 in 2007 at Phnom Dék Health Centre, Rovieng district, Preah Vihear province, and 61 in 2008 at Pramoy Health Centre, Veal Veng district, Pursat province) and 149 patients in Central Tanzania (in 2008 at Kibaoni Health Centre, Kilombero district, Morogoro region).

### Sequencing

Genomic DNA was extracted from 200 μl whole blood using the QIAamp 96 DNA Blood Kit (QIAGEN GmbH, Germany) according to the manufacturer's instructions.

Target sequences in *cyp *and *nat2 *genes of these samples were amplified using PCR. The PCR primers used are listed in Table 1. The amplified regions contained SNPs which are known to alter the function of enzymes involved in the metabolism of anti-malarial drugs (for target loci and effect of the SNP see Table 1). The PCR master mix contained 1 × reaction buffer B (Solis BioDyne, Estonia), 1 × solution S (Solis BioDyne, Estonia), 10 μM of each primer in 1 × Tris-EDTA (see Table 1, Operon Biotechnologies GmbH, Germany), MgCl_2 _(according to Table 1, Solis BioDyne, Estonia), 2 mM of each dNTP in Tris-HCl 10 mM, pH 7.4 (GE Healthcare, Switzerland) and 2 U FIREPol DNA polymerase (Solis BioDyne, Estonia). 1 μl of extracted DNA was mixed with 24 μl of PCR master mix. The PCR protocol was 3 min at 96°C followed by 40 cycles (30 sec at 96°C, 1 min 30 sec at 56-64°C according to Table 1 and 1 min 30 sec at 72°C) with a final elongation for 10 min at 72°C. PCR products were purified and sequenced by Macrogen (Macrogen Ltd., Korea). ABI Prism AutoAssembler version 1.4.0 (Applied Biosystems) was used for assembly and analysis of sequences. The genotype of each patient was then assessed visually. Aliquots of the same PCR products were used for primer extension and microarray analysis.

**Table 1:** Primers used to amplify target sequences in cytochromes P450 isoenzymes and *N*-acetyltransferase 2 genes, MgCl_2 _[μl] for the master mix and annealing temperature [°C] used to amplify target sequence in cytochromes P450 isoenzyme genes and *N*-acetyltransferase 2 genes.

SNP	primer	Sequence	MgCl_2 _[μl]	T [°C]
CYP2A6*2 (479T>A, L160H)	forward reverse	**5'-TCTCTCTCTCTACCTCGACAT-3'** 5'-GTTCCTCGTCCTGGGTGTT-3'	3	64

CYP2B6*5 (1459C>T, R487C)	forward reverse	**5'-CCAGAAGACATCGATCTGAC-3'** 5'-TCTCTCAGAGGCAGGAAGTT-3'	2	64

CYP2B6*6 (516G>T, Q172H)	forward reverse	**5'-TGAGTGATGGCAGACAATCACA-3'** 5'-CAAGTTGAGCATCTTCAGGAACT-3'	2	64

CYP2C8*3 (416G>A, R139K)	forward reverse	**5'-CTAAAGGACTTGGTAGGTGCA-3'** 5'-CAGGATGCGCAATGAAGACC-3'	2	64

CYP2C9*3/*5 (1075A>C, I359L/1080C>G, D360E)	forward reverse	**5'-CTGGTTTATGGCAGTTACACATT-3'** 5'-GAGAAAGTCCAGTTAAACTGCC-3'	2	64

CYP2C19*3 (636G>A, W212X)	forward reverse	**5'-GGGAATTCATAGGTAAGATATTA-3'** 5'-GGAGTGATATAAGCACGCTTTG-3'	2	64

CYP2D6*4 (1846G>A, splicing defect)	forward reverse	**5'-CCGCCTTCGCCAACCACT-3'** 5'-CCCTGCAGAGACTCCTCGGT-3'	2	64

CYP2D6*10 (100C>T, P34S)	forward reverse	**5'-CCCATTTGGTAGTGAGGCAGGT-3'** 5'-CCCCTTCTCAGCCTGGCTTCTTG-3'	2	64

CYP2D6*10 (4180G>C, S486T)	forward reverse	5'-AGCCACCATGGTGTCTTTGCT-3' **5'-TTGCCCTGAGGAGGATGATC-3'**	2	64

CYP2D6*17 (1023C>T, T107I)	forward reverse	**5'-CGCGAGGCGCTGGTGACCAA-3'** 5'-CCAGCTCGGACTACGGTCATCAC-3'	2	64

CYP2D6*17 (2850C>T, R296C)	forward reverse	**5'-GACTCTGTACCTCCTATCCACGTCA-3'** 5'-TCCCTCGGCCCCTGCACTGTTT-3'	2	64

CYP3A4*1B (-392A>G)	forward reverse	**5'-CTCACCTCTGTTCAGGGAAAC-3'** 5'-ATGGCCAAGTCTGGGATGAG-3'	2	64

CYP3A5*3 (6986A>G, splicing defect)	forward reverse	**5'-TGGAGAGTGGCATAGGAGATAC-3'** 5'-CCATACCCCTAGTTGTACGACACA-3'	2.5	64

NAT2*5/*6/*7/*14 (341T>C, I114T/590G>A, R197Q/857G>A, G286E/191G>A, R64Q)	forward reverse	5'-GGGATCATGGACATTGAAGCATATT-3' **5'-ACGTGAGGGTAGAGAGGATATCTG-3'**	2	64

Primers highlighted in **bold **were used for sequencing. SNP positions are indicated in brackets; they were obtained from the Home Page of the Human Cytochrome P450 (CYP) Allele Nomenclature Committee [41], and the Consensus Human Arylamine *N*-Acetyltransferase Gene Nomenclature [44].

### Patient selection for DNA microarray validation

Of 274 patients from Cambodia and Tanzania, 96 were selected (i.e. to fit 96-well plate) for validation of the microarray. Samples were selected by the number of successfully sequenced SNPs and then their ID number. For all 96 selected samples at least 16 out of 18 SNPs have been successfully sequenced.

### Extension control and elimination of non-incorporated nucleotides

As extension control for the microarray, amplified nested PCR product from the *Plasmodium falciparum *chloroquine resistance transporter gene (*pfcrt*) from strains 3D7 (wildtype at loci *pfcrt76 *and *pfcrt97*) and K1 (mutation at locus *pfcrt76 *and wild-type at *pfcrt97*) were mixed at a ratio of 55%:45%. Primers and PCR conditions have been described elsewhere [5].

To eliminate non-incorporated nucleotides prior to primer extension, all nested PCR products of one blood sample were pooled and 10 μl of the pooled PCR products and 0.5 μl of the extension control mix were digested with 8 U shrimp alkaline phosphatase (SAP) and 4 μl 10 × SAP buffer (both Amersham Biosciences, Freiburg, Germany) in a reaction volume of 48 μl for 1 h at 37°C. SAP was inactivated by incubating samples for 15 min at 90°C.

### Primer extension and denaturation

Since the microarray scanner used only supported dual-fluorescence measures simultaneously and because of the large similarity of the *cyp *genes, a strategy of three parallel reactions with different primer and dye combinations had to be applied. Different extension primer mixes (I, II and III) were prepared according to Table 2, each with a total volume of 320 μl containing the corresponding primers (62.5 nM final concentration of each primer, Operon Biotechnologies GmbH, Germany) diluted in 1 × Tris-EDTA (TE). Afterwards, three extension mixes (I, II and III) were prepared (final volume of 8 μl) containing 2 U of HOT TERMIPol^® ^DNA Polymerase (Solis BioDyne, Estonia), 1.8 μl of 10 × Reaction Buffer C (Solis BioDyne, Estonia), 2.5 mM MgCl_2_, and 0.625 μM of the corresponding Cy3- and Cy5-labeled ddNTPs (Perkin Elmer, Schwerzenbach, Switzerland); ddNTP mixes are listed in Table 3. Then, 12 μl SAP digested PCR product of each patient were mixed with 8 μl of the extension mix I, II or III, respectively. The following primer extension protocol was used: 1 min at 94°C followed by 40 cycles of 10 sec at 94°C followed by 40 sec at 50°C. The three mixes of each patient were then pooled again in a 96 well plate and mixed with 10 μl of the hybridization buffer. The hybridization buffer contained 37.5 μM EDTA pH 8.0, 7 pM of two differently labelled positive hybridization controls 5'-GCCTCCACGCACGTTGTGATATGTA- [Cy3]-3' and 5'- CTGTGACAGAGCCAACACGCAGTCT- [Cy5]-5' (Operon Biotechnologies GmbH, Germany), and 3% sodium dodecyl sulphate (SDS). The plate was incubated for 1 min at 94°C and immediately chilled on ice for 2 min.

**Table 2:** Extension primers of three parallel mixes.

SNP	Primer	Mix
CYP2C8*3.1	5'-GGAATTTTGGGATGGGGAAGA-3'	I
CYP2A6*2	5'-GCTTCCTCATCGACGCCC-3'	I
CYP2D6*4	5'-CCGCATCTCCCACCCCCA-3'	I
CYP2D6*10 (100C>T)	5'-ACGCTGGGCTGCACGCTAC-3'	I
CYP2D6*17 (2859C>T)	5'-AGCTTCAATGATGAGAACCTG-3'	I
CYP2D6*17 (1023C>T)	5'-CCCGAAACCCAGGATCTGG-3'	I
CYP3A5*3	5'-TGGTCCAAACAGGGAAGAGATA-3'	I
CYP3A4*1B	5'-CATAAAATCTATTAAATCGCCTCTCTC-3'	I
E. CYP2C9*3	5'-TGCACGAGGTCCAGAGATAC-3'	II
E. CYP2C9*5	5'-CAGGCTGGTGGGGAGAAG-3'	II
E. CYP2B6*6 (516G>T)	5'-AGATGATGTTGGCGGTAATGGA-3'	II
E. CYP2D6*10 (4180G>C)	5'-GTGTCTTTGCTTTCCTGGTGA-3'	II
*pfcrt76*	5'-TTTGTTTAAAGTTCTTTTAGCAAAAATT-3'	II
*pfcrt97*	5'-GTTTTGTAACATCCGAAACTCA-3'	II
NAT2*7	5'-GTGCCCAAACCTGGTGATG-3'	III
NAT2*14	5'-TTGATCACATTGTAAGAAGAAACC-3'	III
CYP2C19*3	5'-AGGATTGTAAGCACCCCCTG-3'	III
CYP2B6*5	5'-TACCCCCAACATACCAGATC-3'	III
NAT2*5	5'-CTTCTCCTGCAGGTGACCA-3'	III
NAT2*6	5'-TATACTTATTTACGCTTGAACCTC-3'	III

**Table 3:** Labelled ddNTPs used for the three parallel extension mixes.

ddNTP and label	Mix
ddATP Cy3	I
ddCTP Cy3	
ddGTP Cy5	
ddUTP Cy5	

ddATP Cy5	II
ddCTP Cy3	
ddGTP Cy5	
ddUTP Cy3	

ddATP Cy3	III
ddCTP Cy5	
ddGTP Cy5	
ddUTP Cy5	

### Microarray production and microarray hybridization

Aldehyde-activated ArrayIt^® ^SuperAldehyde 2 glass slides with SuperMask™ 16 (EBN European Biotech Network, Dolembreux, Belgium) were used. Oligonucleotides (Operon Biotechnologies GmbH, Germany) corresponding to the antisense DNA of the extension primers, extension controls (Table 2) and positive hybridization controls were spotted onto the microarrays in triplicate. The spotting was done by the DNA Array Facility of the Center for Integrative Genomics, University of Lausanne, Switzerland, using solutions of 50 μM oligonucleotide in 180 mM phosphate buffer (pH 8.0). All oligonucleotides had a C7-aminolinker attached to the 3' end. Anchor oligonucleotides prelabeled with Cy3 and Cy5 and four oligonucleotides with a random sequence were added as positive and negative controls, respectively. Of the pooled and denatured primer extension reaction mixture 35 μl were transferred to one well of the microarray, and 6 μl 20 × Standard Saline Citrate (SSC) were added. In each well, representing a single microarray, DNA from one patient was hybridized. Hybridization was carried out in a humid chamber at 50°C for 90 min. After hybridization, the slide was washed at room temperature in 2 × SSC with 0.2% SDS for 10 min, followed by a wash with 2 × SSC for 10 min, and a final wash with 2 × SSC plus 2% ethanol for 2 min. These three steps represent the first wash step. Slides were dried with compressed air.

### Data acquisition

Microarrays were scanned at 635 nm and 532 nm using an Axon 4100A fluorescence scanner (Bucher Biotec AG, Basel, Switzerland). After the first scan slides were washed and scanned again and after each wash, Cy3 and Cy5 images were acquired and analysed using the Axon GenePix Pro software (version 6.0). An in house developed perl script based on Kestler's statistics module [36] was used to call SNPs based on probe signal intensities. The script calculates receiver operating characteristic (ROC) curves using signal intensity values from the set of positive and negative controls for each hybridization. Hybridization specific thresholds that maximize both sensitivity and specificity were then used to make SNP calls.

### Data comparison

SNP data gathered from sequencing and from microarray analysis were compared. Kappa statistics was used as an approach to the evaluation of agreement for the categorical data obtained by the two methods. The kappa index was calculated from contingency tables as follows *κ *= ((a + b) × (a + c) + (c + d) × (b + d))/(a + b + c + d)^2^

The kappa index was interpreted based on the criteria of Landis and Koch [37]. Hardy-Weinberg equilibrium was tested using the chi-square Hardy-Weinberg equilibrium test calculator for bi-allelic markers of the Online Encyclopedia for Genetic Epidemiology studies [38].

### Ethical approval

All the applied protocols were approved by the ethics committee of the two cantons of Basel (Ethikkommission beider Basel, EKBB) and the responsible local authorities (i.e. in Tanzania from the Institutional Review Board of the Ifakara Health Institute and the National Institute for Medical Research Review Board and in Cambodia from the National Ethics Committee for Health Research). Blood samples were collected following written informed consent in the respective local language (Khmer or Swahili) from the participants or their guardians.

## Results

Agreement between results obtained from sequencing and from the microarray on 18 SNPs within eight *cyp *isoenzyme genes and *nat2 genes *from 26 Cambodian and 70 Tanzanian malaria patients was tested. The results are summarized in Table 4. For some SNPs agreement ranged from substantial to almost perfect, whilst for other SNPs a large variability from slight to substantial agreement was found.

**Table 4:** Comparison of SNP data acquired either by sequencing or DNA microarray technology.

		1^st ^wash			2^nd ^wash			
**SNP**	**Country**	***κ***	***n***	**%**	***κ***	***n***	**%**	**Agreement**

CYP2A6*2	Cambodia	0.87	24	88.9	0.92	19	70.4	Substantial to almost
	Tanzania	0.83	58	82.9	0.61	53	75.7	perfect

CYP2B6*5	Cambodia	0.94	27	100.0	0.94	27	100.0	Almost perfect
	Tanzania	0.92	67	95.7	0.86	67	95.7	

CYP2B6*6	Cambodia	0.42	26	96.3	0.42	27	100.0	Fair to moderate
	Tanzania	0.35	63	90.0	0.40	59	84.3	

CYP2C8*3	Cambodia	0.97	20	74.1	1.00	20	74.1	Substantial to almost
	Tanzania	0.90	39	55.7	0.79	38	54.3	perfect

CYP2C9*3	Cambodia	0.92	26	96.3	0.94	26	96.3	Almost perfect
	Tanzania	0.90	67	95.7	0.82	64	91.4	

CYP2C9*5	Cambodia	0.98	27	100.0	1.00	27	100.0	Almost perfect
	Tanzania	0.93	67	95.7	0.86	70	100.0	

CYP2C19*3	Cambodia	1.00	27	100.0	1.00	26	96.3	Almost perfect
	Tanzania	0.90	97	95.7	0.83	66	94.3	

CYP2D6*4	Cambodia	0.98	26	96.3	1.00	26	96.3	Almost perfect
	Tanzania	0.91	63	90.0	0.88	65	92.9	

CYP2D6*10 (100C>T)	Cambodia	0.59	24	88.9	0.64	20	74.1	Moderate to substantial
	Tanzania	0.72	31	44.3	0.76	27	38.6	

CYP2D6*10 (4180G>C)	Cambodia	0,40	21	77.8	0.50	17	63.0	Fair to moderate
	Tanzania	0.45	45	64.3	0.51	38	54.3	

CYP2D6*17 (1023C>T)	Cambodia	0.86	19	70.4	1.00	6	22.2	Moderate to almost
	Tanzania	0.59	37	52.9	0.49	37	52.9	perfect

CYP2D6*17 (2850C>T)	Cambodia	0.29	7	25.9	0.20	6	22.2	Slight to substantial
	Tanzania	0.66	20	28.6	0.59	21	30.0	

CYP3A4*1B	Cambodia	0.00	6	22.2	0.00	5	18.5	Slight to substantial
	Tanzania	0.76	23	32.9	0.69	22	31.4	

CYP3A5*3	Cambodia	0.39	21	77.8	0.42	17	63	Fair to substantial
	Tanzania	0.73	61	87.1	0.65	58	82.9	

NAT2*5	Cambodia	0.32	14	51.9	0.38	8	29.6	Fair to moderate
	Tanzania	0.47	37	52.9	0.53	28	40.0	

NAT2*6	Cambodia	0.70	26	96.3	0.73	25	92.6	Substantial
	Tanzania	0.74	62	88.6	0.71	62	88.6	

NAT2*7	Cambodia	0.72	25	92.6	0.70	22	81.5	Substantial
	Tanzania	0.75	55	78.6	0.67	49	70.0	

NAT2*14	Cambodia	1.00	27	100.0	1.00	27	100.0	Substantial to almost
	Tanzania	0.76	62	88.6	0.74	61	87.1	perfect

Data was acquired in 27 Cambodian and 70 Tanzanian malaria patients. *κ *indicates the kappa index. n is the number and % the percentage of samples with results for both techniques.

Where applicable, Chi-square Hardy-Weinberg equilibrium tests for the allele frequencies acquired by the microarray showed that most SNPs were significantly (*P *< 0.01) out of equilibrium (Table 5). Exceptions were CYP2D6*17 (1023C>T) (*P *= 0.50) for Tanzania after the 1^st ^wash; CYP2C8*3 (*P *= 0.92), CYP2C9*3 (*P *= 0.92), CYP2C9*5 (*P *= 0.92), CYP2D6*4 (*P *= 0.92) and CYP3A5*3 (*P *= 0.16) after the 1^st ^wash and CYP3A5*3 (*P *= 0.01) and *NAT2 *(*P *= 0.07) after the 2^nd ^wash for Cambodia.

**Table 5:** Chi-square Hardy-Weinberg equilibrium tests for SNP data acquired by DNA microarray technology.

	Cambodia				Tanzania			
	**1^st ^wash**		**2^nd ^wash**		**1^st ^wash**		**2^nd ^wash**	

**SNP**	***χ*^2^**	***P***	***χ*^2^**	***P***	***χ*^2^**	***P***	***χ*^2^**	***P***

CYP2A6*2	23.00	< 0.01	7.29	< 0.01	23.57	< 0.01	34.02	< 0.01
CYP2B6*5	N.A.		N.A.		54.39	< 0.01	32.76	< 0.01
CYP2B6*6	N.A.		N.A.		16.90	< 0.01	17.41	< 0.01
CYP2C8*3	0.01	0.92	N.A.		8.22	< 0.01	39.35	< 0.01
CYP2C9*3	0.01	0.92	N.A.		22.08	< 0.01	11.13	< 0.01
CYP2C9*5	0.01	0.92	N.A.		14.69	< 0.01	14.30	< 0.01
CYP2C19*3	N.A.		N.A.		18.22	< 0.01	9.98	< 0.01
CYP2D6*4	0.01	0.92	N.A.		20.87	< 0.01	18.55	< 0.01
CYP2D6*10 (100C>T)	24.00	< 0.01	7.39	< 0.01	31.00	< 0.01	27.00	< 0.01
CYP2D6*10 (4180G>C)	13.67	< 0.01	N.A.		11.94	< 0.01	38.00	< 0.01
CYP2D6*17 (1023C>T)	11.90	< 0.01	7.00	< 0.01	0.45	0.5	35.34	< 0.01
CYP2D6*17 (2850C>T)	7.35	< 0.01	10.00	< 0.01	37.66	< 0.01	30.03	< 0.01
CYP3A4*1B	N.A.		N.A.		N.A.		24.00	< 0.01
CYP3A5*3	1.98	0.16	6.39	0.01	13.36	< 0.01	18.74	< 0.01
N.A.T2*5	8.41	< 0.01	3.32	0.07	13.83	< 0.01	13.70	< 0.01
N.A.T2*6	N.A.		N.A.		33.29	< 0.01	12.04	< 0.01
N.A.T2*7	9.97	< 0.01	8.63	< 0.01	30.21	< 0.01	24.82	< 0.01
N.A.T2*14	N.A.		N.A.		32.08	< 0.01	19.30	< 0.01

*χ*^2 ^indicates the result from the Hardy-Weinberg equilibrium test, *P *the one-tailed *P*-value, and N.A. that Chi-square could not be calculated because the allele frequency was either 0% or 100%.

In contrast, most allele frequencies acquired by sequencing were found to be in Hardy-Weinberg equilibrium (Table 6). However, in the Tanzanian study population CYP2B6*5 and CYP2D6*4 and in the Cambodian study population CYP2B6*5, CYP2C9*3, CYP2D6*10 (4180G>C), and CYP2D6*17 (2850C>T) were found not to have Hardy-Weinberg proportions.

**Table 6:** Chi-square Hardy-Weinberg equilibrium tests for SNP data acquired by sequencing.

	Cambodia		Tanzania	
**SNP**	***χ*^2^**	***P***	***χ*^2^**	***P***

CYP2A6*2	N.A.		N.A.	
CYP2B6*5	10.86	< 0.01	N.A.	
CYP2B6*6	0.28	0.60	1.81	0.18
CYP2C8*3	N.A.		N.A.	
CYP2C9*3	2.94	0.08	N.A.	
CYP2C9*5	N.A.		0.14	0.71
CYP2C19*3	N.A.		N.A.	
CYP2D6*4	N.A.		15.75	< 0.01
CYP2D6*10 (100C>T)	1.39	0.24	1.34	0.25
CYP2D6*10 (4180G>C)	2.01	0.16	1.24	0.27
CYP2D6*17 (1023C>T)	N.A.		0.05	0.82
CYP2D6*17 (2850C>T)	0.24	0.62	4.94	0.03
CYP3A4*1B	0.10	0.75	0.04	0.84
CYP3A5*3	0.64	0.42	0.04	0.84
N.A.T2*5	0.10	0.75	0.01	0.92
N.A.T2*6	0.48	0.49	1.07	0.30
N.A.T2*7	0.01	0.92	0.14	0.71
N.A.T2*14	N.A.		5.02	0.03

*χ*^2 ^indicates the result from the Hardy-Weinberg equilibrium test, *P *the one-tailed *P*-value, and N.A. that Chi-square could not be calculated because the allele frequency was either 0% or 100%.

## Discussion

Comparison of data generated by microarray analysis with sequencing showed that the performance of the DNA microarray is limited. For some SNPs, i.e. CYP2A6*2, CYP2B6*5, CYP2C8*3, CYP2C9*3/*5, CYP2C19*3, CYP2D6*4 and NAT2*6/*7/*14, agreement ranged from substantial to almost perfect (kappa index between 0.61 and 1.00), whilst for other SNPs a large variability from slight to substantial agreement (kappa index between 0.39 and 1.00) was found, e.g. CYP2D6*17 (2850C>T), CYP3A4*1B and CYP3A5*3. No clear trend in rise or decline of agreement between methods was visible from the 1^st ^to the 2^nd ^wash of the slide and thus it remains ambiguous whether repeated washing improves the performance of the microarray. However, agreement tends to be lower among the Cambodian samples. A possible explanation is the strong dependence of kappa on true prevalence of the SNPs [39] and the dissimilar distribution of the SNPs of interest in Cambodians and Tanzanians [40]. Furthermore, among most of these SNPs a considerable number of patient samples failed to yield a signal on the microarray (e.g. CYP2D6*17 (2850C>T) and CYP CYP3A4*1B). The trend was clearly higher in samples from Tanzania, which might also be due to a lower quality of DNA arising from sub-optimal storage conditions after blood withdrawal. Sequencing data agreed with published reference sequences from public sources (Human Cytochrome P450 (*CYP*) Allele Nomenclature Committee [41]. The majority of samples genotyped by sequencing was found to be in Hardy-Weinberg equilibrium (exceptions were CYP2B6*5 in Cambodians and CYP2D6*4 in Tanzanians), proving that sampling was unbiased. The cases where population data generated by microarray was found not to be in Hardy-Weinberg proportion could be attributed to the large number of patient samples that failed to yield a signal on the microarray resulting in smaller simple size.

Because *cyp*s evolved out of a single ancestor [1,42], they show very close sequence similarities, which in turn makes it difficult to design gene specific primers, in particular extension primers that have to be designed at a defined position. It, therefore, became almost impossible to develop a single multiplex PCR and thus the microarray method described here is time consuming and laborious. This is in contrast to a similar microarray developed for the analysis of drug resistance associated SNPs in *P. falciparum *genes that permits the simultaneous analysis of many SNPs in hundreds of samples in a very short time period (approximately 15 h for four 96-well plates) with significantly reduced costs compared to other systems [5].

Furthermore, the costs of sequencing have decreased considerably during the last years and this trend may well continue. On the other hand, the costs of microarray reagents (especially Cy3- and Cy5-labeled ddNTPs that are used in three combinations) and glass slides for arraying have increased and are unlikely to decrease over time. So the costs for the microarray technology are not considerably lower anymore and, therefore, overall costs of both methods have become comparable.

While SNP analysis microarray has been successfully used to analyse point mutations in drug resistance associated genes in *Plasmodium *[5,43], it seems to fail with closely related genes, such as the human *cyp *genes. For the latter, sequencing appears to be a more reliable method.

## Conclusion

Although microarray allows the simultaneous determination of many SNPs, the lack of robustness for the described approach here prohibits its wide use in pharmacogenetics and sequencing occurs to be the more reliable technique. With the availability of large sequencing capacities worldwide, molecular-epidemiological studies using sequencing for a limited number of SNPs in *CYP *genes in a large population are feasible.

## Abbreviations

Cy: Cyanine; *CYP*: Cytochrome P450; ddNTP: Dideoxynucleotide; DNA: Deoxyribonucleic acid; dNTP: Deoxynucleotide; EDTA: Ethylenediaminetetraacetic acid; *NAT2*: *N*-acetyletransferase-2; PCR: Polymerase chain reaction; PFCRT: *Plasmodium falciparum *chloroquine resistance transporter; RFLP: Restriction fragment length polymorphism; ROC: Receiver operating characteristic; SAP: Shrimp alkaline phosphatise; SDS: Sodium dodecyl sulphate; SNP: Single nucleotide polymorphism; SSC: Standard saline citrate; TE: Tris-EDTA

## Competing interests

The authors declare that they have no competing interests. Support for this work was provided by grant No 320000-112479 from the Swiss National Science Foundation.

## Authors' contributions

EMH and SL were involved in microarray technology development and validation, data acquisition and analysis, and writing of the manuscript. WQ has developed the programme for the ROC analysis. FA was involved in the technology development. BG was involved in the overall pharmacogenetic project design and supervision. HPB was involved in the project design, technology development and in writing the manuscript
